# A macrophage-cell model of HIV latency reveals the unusual importance of the bromodomain axis

**DOI:** 10.1186/s12985-024-02343-9

**Published:** 2024-04-05

**Authors:** Javan K. Kisaka, Daniel Rauch, Malachi Griffith, George B. Kyei

**Affiliations:** 1 Department of Medicine, Washington University School of Medicine in St. Louis, St. Louis, MO 63110 USA; 2 McDonnell Genome Institute, Washington University School of Medicine in St. Louis, St. Louis, MO 63108 USA; 3 Department of Molecular Microbiology, Washington University School of Medicine in St. Louis, St. Louis, MO 63110 USA; 4 Department of Virology, College of Health Sciences, Noguchi Memorial Institute for Medical Research, University of Ghana, Accra, Ghana; 5 Medical and Scientific Research Center, University of Ghana Medical Center, Accra, Ghana

**Keywords:** HIV, Macrophages, Latency, Latency-reversing agents, THP-1, Cell line model

## Abstract

**Background:**

Although macrophages are now recognized as an essential part of the HIV latent reservoir, whether and how viral latency is established and reactivated in these cell types is poorly understood. To understand the fundamental mechanisms of viral latency in macrophages, there is an urgent need to develop latency models amenable to genetic manipulations and screening for appropriate latency-reversing agents (LRAs). Given that differentiated THP-1 cells resemble monocyte-derived macrophages in HIV replication mechanisms, we set out to establish a macrophage cell model for HIV latency using THP-1 cells.

**Methods:**

We created single-cell clones of THP-1 cells infected with a single copy of the dual-labeled HIV_GKO_ in which a codon switched eGFP (csGFP) is under the control of the HIV-1 5’ LTR promoter, and a monomeric Kusabira orange 2 (mKO2) under the control of cellular elongation factor one alpha promoter (EF1α). Latently infected cells are csGFP^−^, mKO2^+,^ while cells with actively replicating HIV (or reactivated virus) are csGFP^+^,mKO2^+^. After sorting for latently infected cells, each of the THP-1 clones with unique integration sites for HIV was differentiated into macrophage-like cells with phorbol 12-myristate 13-acetate (PMA) and treated with established LRAs to stimulate HIV reactivation. Monocyte-derived macrophages (MDMs) harboring single copies of HIV_GKO_ were used to confirm our findings.

**Results:**

We obtained clones of THP-1 cells with latently infected HIV with unique integration sites. When the differentiated THP-1 or primary MDMs cells were treated with various LRAs, the bromodomain inhibitors JQ1 and I-BET151 were the most potent compounds. Knockdown of BRD4, the target of JQ1, resulted in increased reactivation, thus confirming the pharmacological effect. The DYRK1A inhibitor Harmine and lipopolysaccharide (LPS) also showed significant reactivation across all three MDM donors. Remarkably, LRAs like PMA/ionomycin, bryostatin-1, and histone deacetylase inhibitors known to potently reactivate latent HIV in CD4 + T cells showed little activity in macrophages.

**Conclusions:**

Our results indicate that this model could be used to screen for appropriate LRAs for macrophages and show that HIV latency and reactivation mechanisms in macrophages may be distinct from those of CD4 + T cells.

**Supplementary Information:**

The online version contains supplementary material available at 10.1186/s12985-024-02343-9.

## Background

Advances in antiretroviral therapy (ART) have substantially improved the health of people living with HIV (PLWH) because ART effectively suppresses HIV replication in actively dividing cells. However, ART cannot eliminate proviral HIV that is integrated into latently infected cellular reservoirs such as resting CD4^+^T cells and macrophages [1]. These HIV reservoirs cause rapid viral rebound when ART is interrupted, thus requiring PLWH to maintain life-long therapy. Since antiretroviral therapy faces challenges such as non-adherence, medication side effects, economic costs, and drug resistance, there is an urgent need to find viable lasting options that can lead to sustained viral suppression or elimination [2].

The mechanisms through which HIV establishes and maintains its reservoir in latently infected cells are not fully understood. It is thought that resting CD4^+^T cells [1, 3], residual viral replication [4] as well as sanctuary sites in tissue compartments [5] contribute to the HIV reservoir. Several studies have also shown evidence that proviral clonal expansion of integrated HIV in CD4^+^ T cells [6–8] could be responsible for the viral rebound. Infected CD4^+^T may establish latency as they transition to the resting memory state [9] and persist as long-lived cells with integrated HIV genome [10], resulting in persistent HIV in resting cells with transcriptionally silent viral DNA that cannot be reached by ART [11, 12]. It is the cellular reservoir that HIV cure approaches seek to target.

There are several approaches to HIV cure including shock and kill to reactivate the latent provirus, block and lock which seeks to prevent reactivation, immunotherapy such as broadly neutralizing antibodies and chimeric antigen receptors (CAR) T cells, and gene therapy such as CRISPR/Cas9 to excise or disable the virus in the host genome. Of these, the shock and kill approach is the most studied with several completed and ongoing clinical trials. The approach involves the use of latency-reversing agents (LRAs) to activate viral transcription, protein expression, and virus production in latently infected cells (shock) which could then be eliminated (killed) by immune-mediated clearance or cytolysis [13, 14]. LRAs such as interleukin 2 (IL-2) [15], and anti-CD3 antibody [16] were initially used but had intolerable side effects. Later on, histone deacetylase (HDAC) inhibitors, protein kinase C agonists like bryostatin-1 and its analogs, bromodomain inhibitors, and many others have been tried in cell models and some clinical studies [17–19].

Most HIV reservoir studies are focused on T cells, but it is becoming increasingly clear that macrophages are an important HIV reservoir because they reside in many tissues, are long-lived, and can self-renew. Like T cells, macrophages that are latently infected with HIV can produce virus when reactivated [20]. It has been shown that circulating monocytes [21], microglia, and brain astrocytes [22] harboring viral DNA persist in patients on ART [23]. Therefore, cells from the monocytes/macrophage lineages may play a critical role in the formation and persistence of the HIV reservoir [24–26]. Circulating monocytes can develop into mature macrophages upon migration to tissues [27–29]. Macrophages both in lymphoid and non-lymphoid tissues such as vaginal mucosa [30], urethra [31, 32], and duodenal tissue [33] harbor HIV-1 DNA. However, it is not known how and if macrophages establish HIV latency and how they respond to latency-reversing agents, questions that we can begin to answer with appropriate macrophage models.

The development of HIV-1-infected cell line models has greatly advanced our understanding of the basic mechanisms of HIV-1 latency, as well as how the latent reservoir responds to latency- reversing agents [34]. These cell lines differ in parental cell origin and replication capacity of the provirus. For instance, U1 cells are U937 pro-monocytic cells, ACH-2 and 8E5 are A3.01 sub-clone of CEM T cells while J-Lat is Jurkat T cells. U1 and ACH-2 have replication-competentt HIV with mutations in Tat and TAR respectively while the J-Lat cell lines have one copy of HIV∆envGFP inserted [35, 36]. In addition to cell line models, several primary CD4^+^T cell models have also been described [37–41]. However, while there are several models of HIV latency for T cells, similar models for macrophages are just beginning to get attention. Wong, M. E et al. recently described a model for the quantification of viral reactivation in HIV-infected human monocyte-derived macrophages (MDM) [42]. However, in this mode, there is s significant loss of adherence of reactivated cells which could underestimate the percentage of reactivated cells. On the other hand, the tendency of MDM to form multinuclear cells could overestimate cell input versus proportion of latently infected cells. In addition, primary cell models are less amenable to genetic manipulations to uncover the mechanisms of latency in macrophages.

In this work we have developed a macrophage model for HIV latency using the macrophage-like cell line THP-1 cells. We took advantage of the fact that during HIV replication, THP-1 cells behave like dividing cells in their monocytic state and take on the characteristics of macrophages when terminally differentiated by phorbol 12-myristate 13-acetate (PMA). These features have been confirmed in studies with SAMHD1, cyclin L2, and DYRK1A whereby these proteins do not affect HIV in dividing cells (including THP-1) but have profound effects in primary macrophages and differentiated THP-1 cells [43, 44]. Therefore, we set out to create a macrophage model for HIV latency using THP-1 cells since they can be differentiated into macrophage-like cells and have shown concordance with primary macrophages in multiple systems. A cell line macrophage model will allow for genetic manipulations on a large scale which can then be confirmed in primary macrophage systems. Using different markers for latency and reactivation, latently infected cells were sorted into single clones, expanded and characterized for full length viral integration with one viral copy per cells. Established cell lines were reactivated with a variety of known LRAs following differentiation with PMA. Out of the multiple LRAs tested, the Bromodomain and Extraterminal bromodomain inhibitors (BETi), JQ1, and I-BET-151 were the LRAs that most significantly induced HIV reactivation in latently infected differentiated THP-1 cells, finding that was confirmed in primary macrophages.

## Methods

### Cell culture, reagents, and antibodies

The 293T cells were maintained in Dulbecco’s modified Eagle’s medium (DMEM) supplemented with antibiotic, antimycotic, glutamine, 10% fetal bovine serum (FBS), and sodium pyruvate. THP-1 cells were maintained in RPMI supplemented with L-glutamine and 10% FBS, antibiotic antimycotic, and sodium pyruvate. To differentiate THP-1 cells into macrophages, 10 ng/ml phorbol 12-myristate 13-acetate (PMA) was added to THP-1 in 6 well plates for 48 h. Both 293T and THP-1 cells were obtained from American Type Culture Collection (ATCC). Human monocyte derived macrophages (MDMs) were prepared from peripheral blood mononuclear cells (PBMC) of HIV-1 negative donors purified by Ficoll-Paque density gradient centrifugation (GE Healthcare) followed by lymphocytes removal using EasySep Human Monocytes Enrichment according to manufacturer’s protocol (STEMCELL catalog # 19,059). The isolated monocytes were maintained in RPMI supplemented with L-glutamine and 10% FBS, antibiotic, antimycotic and sodium pyruvate then differentiated with 50 ng/ml of M-CSF for 7 days before transfection. Cells were treated with varying concentrations of LRAs for 48 h: 100nM SDL148, 100nM SDL146 [45], 1nM Bryostatin-1 (Catalog # 20-381-110UG) and 5µM Harmine (Catalog # 507550R) obtained from Fisher Scientific, 10ng/ml TNF-α (Catalog # T6674), 1-5µM JQ1 (Catalog # SML1524-5MG), 1µM SAHA (Catalog # SML0061), 0.5µM i-BET151 (Catalog # GSK1210151A) and 10ng/ml PMA (Catalog # 524400-25MG) obtained from Sigma, 2 µl/ml PMA/Ionomycin (Catalog # 00-4970-93) was obtained from eBioScience. Fluorescence-activated cell sorter (FACS) analysis was performed for GFP-positive cells using the Becton Dickinson (BD) FACS. Analysis was done using BD’s CellQuest Pro software. THP-1 cells with BDR4 knockdown were maintained in complete RPMI as described above. Primary antibodies of rabbit polyclonal BRD4 (Catalog # sc-48,772) and rabbit polyclonal beta-actin (sc-130,656), were purchased from Santa Cruz Biotechnologies. NSD1 (Catalog # MA515420) and UNKL (Catalog # 15000-1-AP) antibodies were from Thermofisher. Cyclin L2 antibody was obtained from Novus Biologicals (Catalog # NB100-87009). Secondary antibodies goat anti-mouse and goat anti-rabbit were purchased from Invitrogen.

### Plasmids, siRNA transfections, and western blots

The pool of BRD4 siRNA (smart pool) was obtained from Santacruz with catalog number sc-43,639. The HIV_GKO_ plasmid was a gift from Eric Verdin (Addgene plasmid # 112,234). HIV_GKO_ and VSVG plasmid was used to transfect 293T cells using lipofectamine 3000 transfection reagent (catalog number: L3000001) according to the manufacturer’s protocol. For siRNA-mediated knockdown of BRD4, differentiated THP-1 was transfected by nucleoporation using Lonza nucleofector according to the manufacturer’s protocol. Forty-eight hours post-transfection, cells were washed twice with 1X PBS and lysed with 1X PBS buffer with 0.2% NP40 and protease inhibitor cocktail (Roche). Total protein was measured with a BCA protein assay kit. Protein samples were separated on a 10% SDS-PAGE and transferred to nitrocellulose. The membrane was blocked for 1 h at room temperature in 4% BSA in PBS, 0.05% Tween 20 followed by incubation with primary antibodies overnight at 4 °C. The membrane was washed with 1X PBS, 0.05% Tween 20, and probed with HRP-conjugated secondary antibodies at room temperature for 1 h. The membranes were then stained with Immobilon Western Chemiluminescent HRP (Millipore).

### Fluorescence microscopy

Differentiated THP-1 cells harboring reactivated latent HIV-1 cultured on coverslips were rinsed twice with 1X PBS, fixed with 4% paraformaldehyde for 10 min, and then washed twice with 1X PBS. Images were taken and processed with fluorescence microscopy. (Nikon Eclipse Ti-E inverted microscope)

### Cell lines, virus production, infections, and cell sorting

HIV_GKO_ virus particles were obtained by co-transfecting 293Tcells using Lipofectamine 3000 reagent according to the manufacturer’s protocol (Life Technologies) with HIV_GKO_ and VSV-G at a ratio of 3:1. Media was changed at 6 h post-transfection and the supernatant containing the viral particles was collected after 24–48 h. The supernatant was cleared by centrifugation (2000 rpm, for 10 min at room temperature) and filtered through a 0.45 µM membrane. Cleared supernatants were then concentrated by ultracentrifugation for 2 h (22,000 g, 2 h, 4 °C). The viral particles were then resuspended in media and stored at − 80 °C till use. Viral concentration was determined by p24 ELISA. Thawed viral particles were used to transduce THP-1 cells to obtain stable cell lines with integrated HIV-1, followed by selection by MKO2 and GFP fluorescence-activated cell sorting. Sorting of infected cells was performed with a FACS Arial II based on their MKO2 and GFP fluorescence markers 4–5 days post-transduction and returned in culture. To obtain the cell lines, transduced undifferentiated THP-1 cells were sorted into 96 well plates at one cell per well followed by 4 weeks of culture as clones expanded. Sample cell clones were then taken for further screening for full length and one viral copy per cell. Cell clones with a single, full-length provirus were expanded further for characterization.

### Treatment with latency-reversing agents and flow cytometry

Monocyte-derived macrophages, differentiated or undifferentiated THP-1 cells were incubated with the various LRAs at the indicated concentrations for 48 h. Cells were trypsinized where indicated, washed, and suspended in 1% paraformaldehyde. FACS was performed using Becton Dickinson (BD) FACS instrument based on the GFP fluorescence markers.

### RNA isolation, quantitative reverse transcriptase PCR (qRT-PCR), and RNA seq

Latent cells treated with LRAs or DMSO control were trypsinized and washed with PBS. Total RNA was isolated with an RNeasy mini kit (Qiagen catalog # 74,104) according to the manufacturer’s protocol. Real-time qRT-PCR was performed with Bio-Rad One-Step RT-PCR KIT (Catalog number 1,725,150) according to the manufacturer’s protocol using the Bio-Rad CFX Connect system with SYBR green. Gene expression was calculated by the comparative threshold cycle method (2^−ΔΔ*CT*^) using GAPDH as a control. The primers used are listed in Table S2. For RNA-sequencing, the quality of RNA was examined using Agilent Bioanalyzer. RNA sequencing, and library preparation were performed at Washington University GTAC center.

### Droplet digital PCR (ddPCR) and viral copy number determination

Genomic DNA was extracted from cells using DNeasy Blood & Tissue Kit from Qiagen (Catalog number 69504) and 20 µl of a PCR reaction containing template DNA, HIV 5’ LTR primers, probe labeled with FAM, RPP30 primers, probe labeled with VIC as internal control, and Bio-Rad ddPCR supermix. Droplets were generated with a QX200 Droplet generator and transferred to a 96-well plate. ddPCR was performed with C1000 Touch™ Thermal Cycler with the following cycling conditions: 10 min at 95 °C, 40 cycles each consisting of a 30-second denaturation at 94 °C followed by a 58 °C extension for 60 s, and a final 10 min at 98 °C. Following PCR amplification, ddPCR was analyzed with QX200 Droplet Reader, and data was analyzed using QuantaSoft Software. Because the DNA distribution into the droplets follows a random pattern, each contains either a few or no target sequences and the droplets are clustered into four groups. The QuantaSoft software measures the absolute numbers of droplets that are positive and negative for each fluorophore (FAM and VIC) in a sample. The fraction of positive droplets is then fitted to a Poisson distribution to determine the absolute initial copy number of the target DNA The outcome of this protocol obtained HIV DNA copy numbers normalized to internal cellular input of RPP30 as housekeeping gene. This provides information on the abundance of HIV DNA for accurate determination of viral copy number by comparing the ratios of LTR to internal control RPP30 DNA. Since each cell has two copies of RPP30, a high ratio of HIV DNA to RPP30 (> 0.5) indicates that the proviral copy number is greater than one per cell. The primers used are listed in Table S2.

### Amplification of integration sites

The amplification and determination of the integration sites were performed according to the protocol for mapping integration sites of HIV-1 and other retroviruses adopted from Well, D.W et al [46] and Firouzi, S. et al. [47] using adaptor/linker-mediated amplification of HIV integration sites and bioinformatics pipeline. Briefly, genomic DNA (10 µg DNA in 100 µL MQ) extracted from the respective clones was sheared by sonication using Covaris™ S220 System equipment. The ends of the sheared DNA were repaired and a single dA was added to allow selective linker ligation. This allows the amplification of fragments that contain ends of viral DNA and host segments. Nested PCR was performed between the end of HIV-1 long terminal repeat and the linker for Illumina sequencing. A paired-end read (read 1 and read 2) with a tag read was then acquired with Illumina genome analyzer II. The reads were subsequently mapped against the human genome to determine the proviral insertion sites. A detailed schematic overview is provided in Figure S3.

## Results

### Establishment of HIV latency in a macrophage-like THP-1 cell clone

**Fig. 1:**
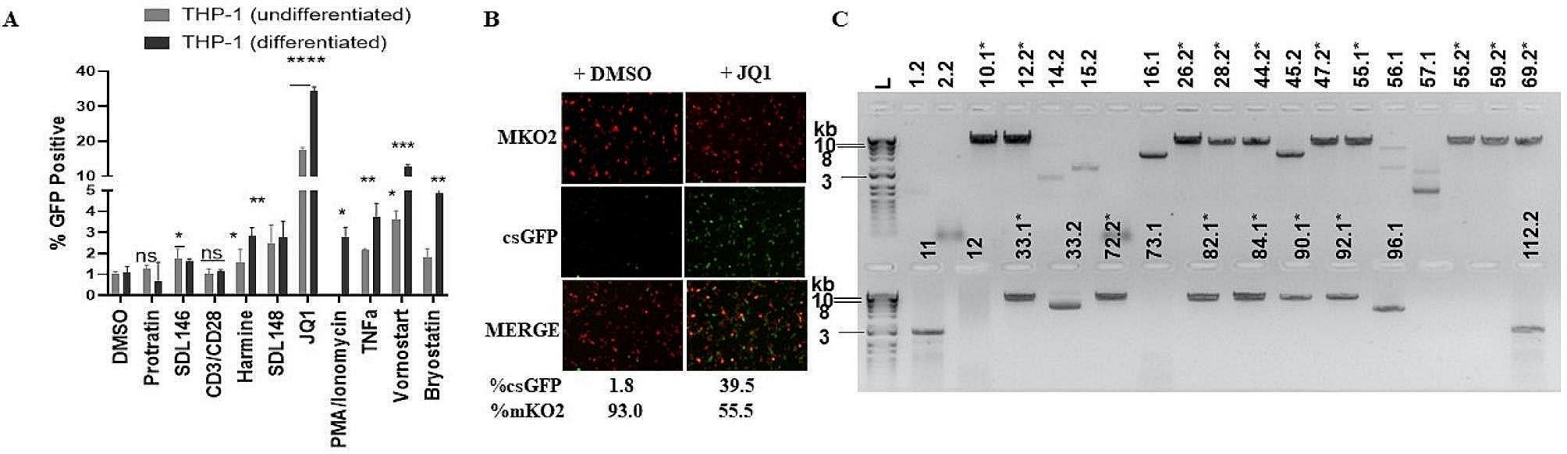
Establishment of HIV latency in macrophage-like THP-1 cell line. (**A**) Screening of latency-reversing agents in latently infected THP-1. Initial screening of LRAs in a mixture of latently infected non-clonal THP-1 cells. (**B**) Reactivation of HIV in latently infected non-clonal dTHP-1 cells. uTHP-1 cells were infected with the HIV-1 reporter virus (HIVGKO) for 4 days and sorted for cells expressing mKO2 (latently infected cells). Cells were transformed into macrophage-like cells by differentiation with 10 ng/ml PMA for 48 hours followed by reactivation with JQ1 (1000 nM) for 48 hours. Cells were then fixed and observed under a fluorescent microscope. Cells with the reactivated virus show csGFP. Percentages show mKO2 + cells that also became csGFP + after reactivation (csGFP+, mKO2+). (**C**) PCR of DNA extracted from expanded single clones with primers covering HIV-1 5’ LTR and envelope to further screen for clones with full-length viral DNA. Clones with full-length single viral copies of integrated viral DNA are shown with asterisks (*). These clones were selected for further characterization. Data are means and error bars indicate ± SEM (*n* = 3). *p < 0.5, *p* < 0.1, **, *p* < 0.01; ***, *p* < 0.001, ns, not significant, Student’s t test

To study HIV-1 latency in the differentiated THP-1 cell line, we obtained a dual-labeled (HIV_GKO_) plasmid developed by Battivelli et al [41] in which a codon switched eGFP (csGFP) is under the control of the HIV-1 5’ LTR promoter and orange fluorescent protein MKO2 is under the control of cellular elongation factor one alpha promoter (EF1α), (Addgene plasmid #112,234). HIV_GKO_ construct has been used in other latency models in primary cell lines and has been demonstrated by others that mKO2-single positive cells represent transcriptionally inactive integrated HIV [41, 48–50]. The different promoters ensure that cells with HIV_GKO_ integrated into their genome show orange fluorescence (shown as red) while those actively expressing HIV show green fluorescence. Expression of mKO2 indicates that integrated HIV is present in the cell. Cells with integrated HIV undergoing active viral transcription are under HIV LTR promoter and will fluorescence both csGFP and mKO2. When the HIV LTR promoter is inactive (latent), cells will not express viral genes or albeit minimally. These cells will be identified as mKO2 single-positive-cells and distinguishes latently infected cells from those productively infected (csGFP and mKO2 double positive) and non-infected (double negative). To perform an initial test of this system, we infected undifferentiated THP-1 (uTHP-1) cells at a low multiplicity of infection (m.o i) of 0.5 with VSVG pseudotyped HIV_GKO_ for 4–5 days. Using a cell sorter, a small population of latently infected cells expressing low mKO2 (2–4%) were sorted from productively infected cells expressing csGFP (5–10%). The mKO2-expressing sorted cells, considered as latently harboring HIV were treated with 10 ng/ml PMA for 48 h to transform them into differentiated THP-1 cells (dTHP-1) which mimics resting macrophages (M0) [51–53]. Addition of PMA did had no effect on the transition from mKO2 to csGFP. Next, we treated the uTHP-1 and the dTHP-1 cells for 48 h with well-established LRAs with different mechanisms of action including CD3/CD28 (as a control since THP-1 cells have no T cell receptors), HDAC inhibitor Vorinostat, PMA/Ionomycin, bryostatin-1, tumor necrosis factor-alpha (TNF-α), and the bromodomain inhibitor JQ1 to determine their ability to induce HIV expression. Out of these LRAs, JQ1 showed the most significant expression of GFP (up to 35%) in dTHP-1 cells as measured by FACS (Fig. 1A) in this non-clonal mixture of latently infected cells. Examination of JQ1-treated non-clonal dTHP-1 cells with flourescent microscopy showed HIV reactivation (mKO2^+^, csGFP^−^ to mKO2^+^, csGFP^+^) confirming the FACS results (Fig. 1B), indicating that HIV could be shocked out of latency in dTHP-1 cells. Since this initial sorting of ‘global latent cells’ may contain more than one viral copy, we next sought to use uTHP-1 cells to generate a clonal cell line, with each cell harboring a single copy of full-length latent HIV_GKO_. For this, we infected uTHP-1 cells with HIV_GKO_ at 0.1–0.5 m.o.i for 4–5 days and sorted cells with low mKO2 expression using the gating strategy shown in Fig. S1. Using a cell sorter, the low mKO2 expressing cells were distributed into 96 well plates, one cell per well, followed by single-cell expansion in culture. The single expanded clones were then subjected to secondary screening by microscopy and flow cytometry analysis of each clone and only mKO2^+^ single positive clones were selected for further characterization. This diminished the possibility of residual HIV-expressing cells being carried over in each established latent clone. We isolated genomic DNA from 60 different expanded single clones and determined viral copy number by Bio-Rad QX200 droplet PCR using an HIV 5’ LTR probe and RPP30 as a housekeeping gene (Fig. S2). Out of the 60 screened clones, 19 had one copy of the HIV provirus per cell. For the 19 potential clones, we determined the ones with full-length HIV by performing PCR with primers covering HIV 5’ LTR and envelope. From the above processes, we selected 10 different clones with full-length HIV for further analyses. As shown with representative gels in Fig. 1C, we found that latently infected cells harbor a mixture of integrated viral DNA with either deleted, partial (#1.2, 2.2, 14.2, 15.2,16.1, 45.2, 11, 12, and 112.2), or full-length HIV proviruses (marked with asterisk * in Fig. 1C). We selected the clones with single copy full-length integrated viral DNA to determine the integration sites using the methods illustrated in Fig. S3. When we identified the integration sites for six major clones, they were found in actively transcribed genes such as CCNL2, NSD1, UNKL, and RERE, all of which were expressed in macrophages as determined by RNAseq data and Western blots (Fig. S4).

**Fig. 2:**
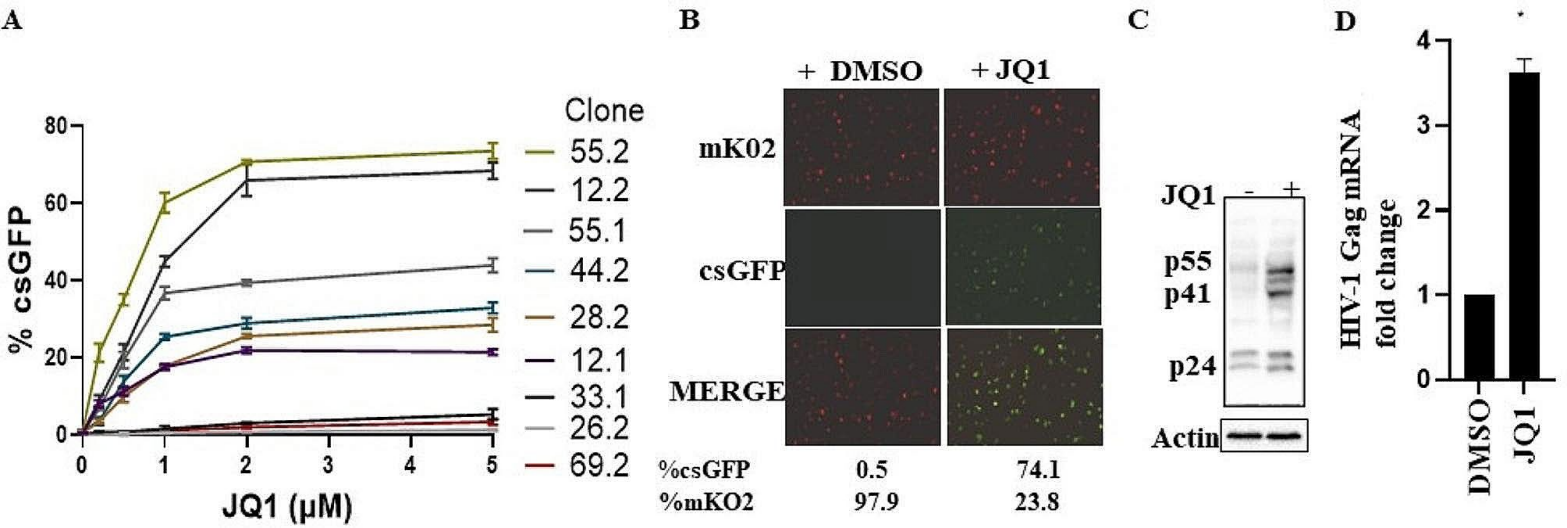
Reactivation of latently infected dTHP-1 single clones with JQ1. (**A**) Expression of csGFP in different clones harboring one copy of HIV. Established clones were differentiated for 48 h followed by incubation with JQ1, and csGFP (reactivation) was measured with FACS analysis. (**B-D**) A full range of HIV products is expressed upon reactivation of latent HIV in representative clone 55.2. Differentiated cells were reactivated with JQ1 for 48 h and HIV expression was assayed with microscopy (**B**), Western blot for HIV proteins (**C**), and quantitative RT PCR for gag mRNA (**D**). Percent of csGFP + cells is shown below the image in B. Data are means, and error bars indicate SEM (*n = 3*). *, *p* < 0.001, Student’s t test

Next, we sought to determine whether JQ1 can reactivate different clonally expanded differentiated THP-1 cells harboring a single copy of HIV. First, using flow cytometry, we measured sensitivity for viral reactivation in nine clones and found variable reactivation with increasing concentrations of JQ1 (Fig. 2A, Table S1). While clones such as 12.2 and 55.2 showed reactivation of 60–75% (mKO2+, csGFP+), others like 26.2 and 33.1 showed virtually no reactivation. Thus, we observed that single clones varied from minimal to massive reactivation, compared to the mixture of latently infected cells which showed moderate reactivation (Figs. 1A and 2A. Second, we used microscopy to visualize the reactivation for 55.2, the clone most responsive to JQ1. Compared to the non-clonal “global” population of mKO2 + latent cells in Fig. 1B, clone 55. 2 had no significant csGFP basal expression (0.5%) before latency reversal (Fig. 2B). Third, western blot and qRTPCR targeting gag showed significant increases in HIV protein and RNA products when differentiated clone 55.2 cells were treated with JQ1 (Fig. 2C and D). However, the proportional increases in the protein and RNA levels upon reactivation were much smaller compared to the csGFP. Taken together, the results show that we have successfully established latent THP-1 single clones with full-length, integrated HIV provirus that can be differentially reactivated in response to JQ1.

### Lagency reversal using different LRAs in THP-1 cells and primary macrophages

**Fig. 3:**
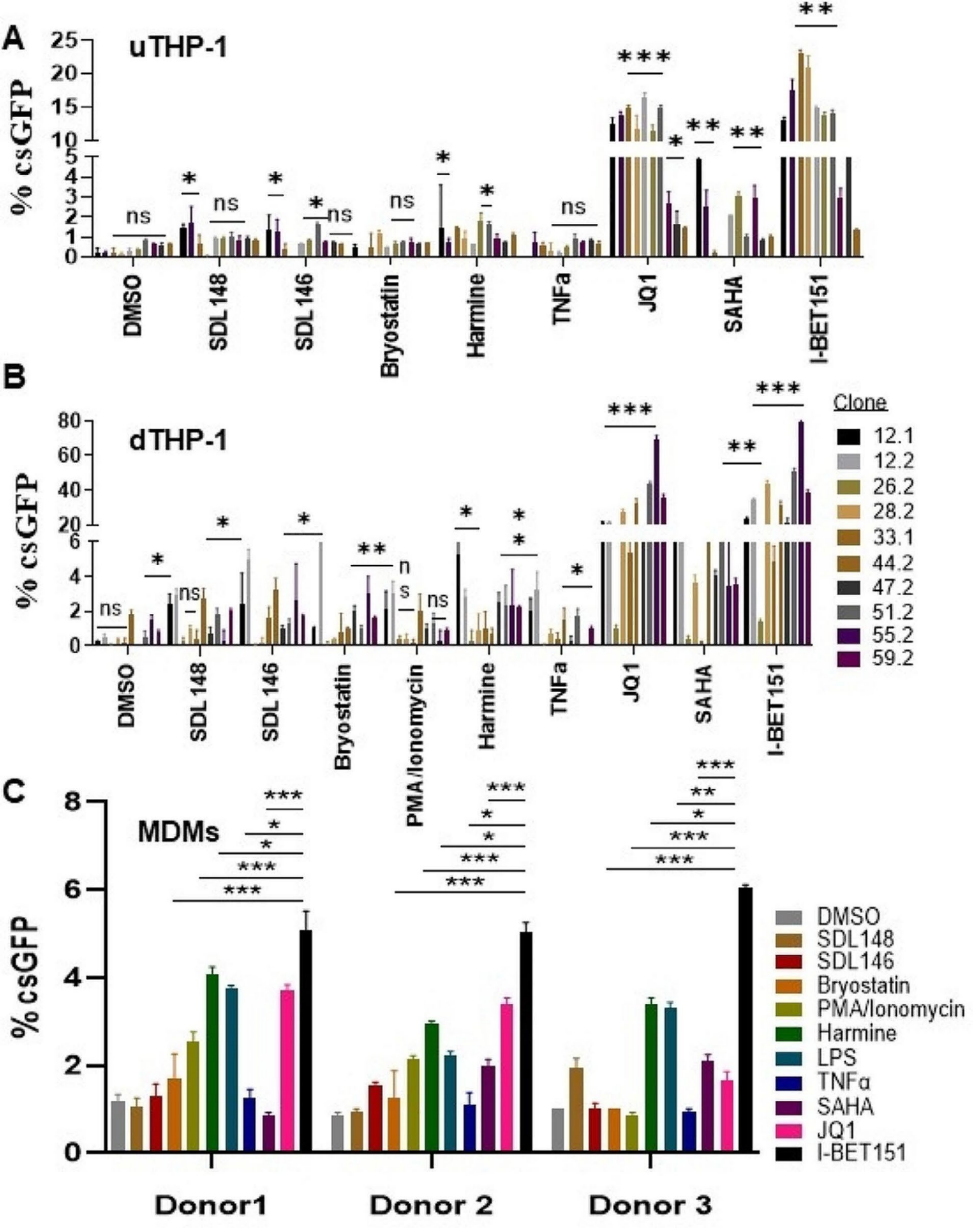
Latency reversal in the established cell lines and MDMs using different latency-reversing agents. (**A, B**) Latency reversal was more efficient in dTHP-1 cells for all the LRAs that could reactivate HIV from latency. Ten different clones with latent HIV were reactivated with indicated LRAs for 48 h in uTHP-1 (**A**) or dTHP-1 cells (**B**). HIV reactivation from latency was measured with FACS of csGFP-expressing cells. (**C**) Latently infected MDMs can be reactivated from latency. MDMs from three healthy donors were infected with HIV_GKO_ and sorted for latently infected mKO2-expressing cells. Cells were incubated with the indicated LRAs for 48 and reactivation was measured with csGFP FACS as before. Data are means and error bars indicate ± SEM (*n = 3*). *, *p* < 0.05, **, *p* < 0.001; ***, *p* < 0.0001, ns, not significant, Student’s t test

With this new HIV latency/reactivation model, we compared the reactivation potential of other LRAs in uTHP-1 cells (Fig. 3A) and those differentiated with 10 ng/ml PMA for 48 before LRA treatment (Fig. 3B). We found that while bromodomain inhibitors JQ1 and I-BET151 had substantial reactivation effects on the majority of the clones, LRA’s such as TNF-α, HDAC inhibitors (SAHA, SDL148, and SDL146) and bryostatin-1 had minimal effect on HIV-1 reactivation. Overall, reactivation in uTHP-1 cells was much lower (up to 20%) compared to dTHP-1 (up to 74%). The reactivation by JQ1 and I-BET151 was 3 to 5-fold higher when THP-1 was differentiated into macrophages. The most responsive clone was 55.2 while clone 26.2 was insensitive to the LRAs tested both in uTHP-1 and dTHP-1 cells. Next, we determined if the LRAs behaved similarly in primary monocyte-derived macrophages (MDMs). The isolated MDMs were infected with the HIV_GKO_ for 5–6 days at m.o.i of 0.5-1.0 and single copy viral insertion was confirmed with droplet digital PCR. After sorting for latently infected cells (gating strategy shown in Figure S5), we added LRAs for 48 h, and reactivation levels were measured as a percentage of csGFP-positive cells using flow cytometry. There was reduced reactivation in MDMs compared with dTHP-1 cells (Fig. 3C). I-BET151 performed the best among the different LRAs and was consistent across all three donors. The other bromodomain inhibitor JQ1 resulted in reactivation in two donors but was marginal in the third donor. The DYRK1A inhibitor Harmine and lipopolysaccharide (LPS) also showed significant reactivation across all three donors. The difference in reactivation between I-BET151 and the other responsive LRAs was statistically significant (Fig. 3C). Remarkably, potent, and well-known LRAs like bryostatin-1, SAHA, and PMA/Ionomycin had no effect in MDMs. Taken together, the data shows that dTHP-1 cells recapitulate reactivation outcomes observed in MDMs, especially for the bromodomain inhibitors.

### Knockdown of BRD4 reactivates latent HIV

**Fig. 4:**
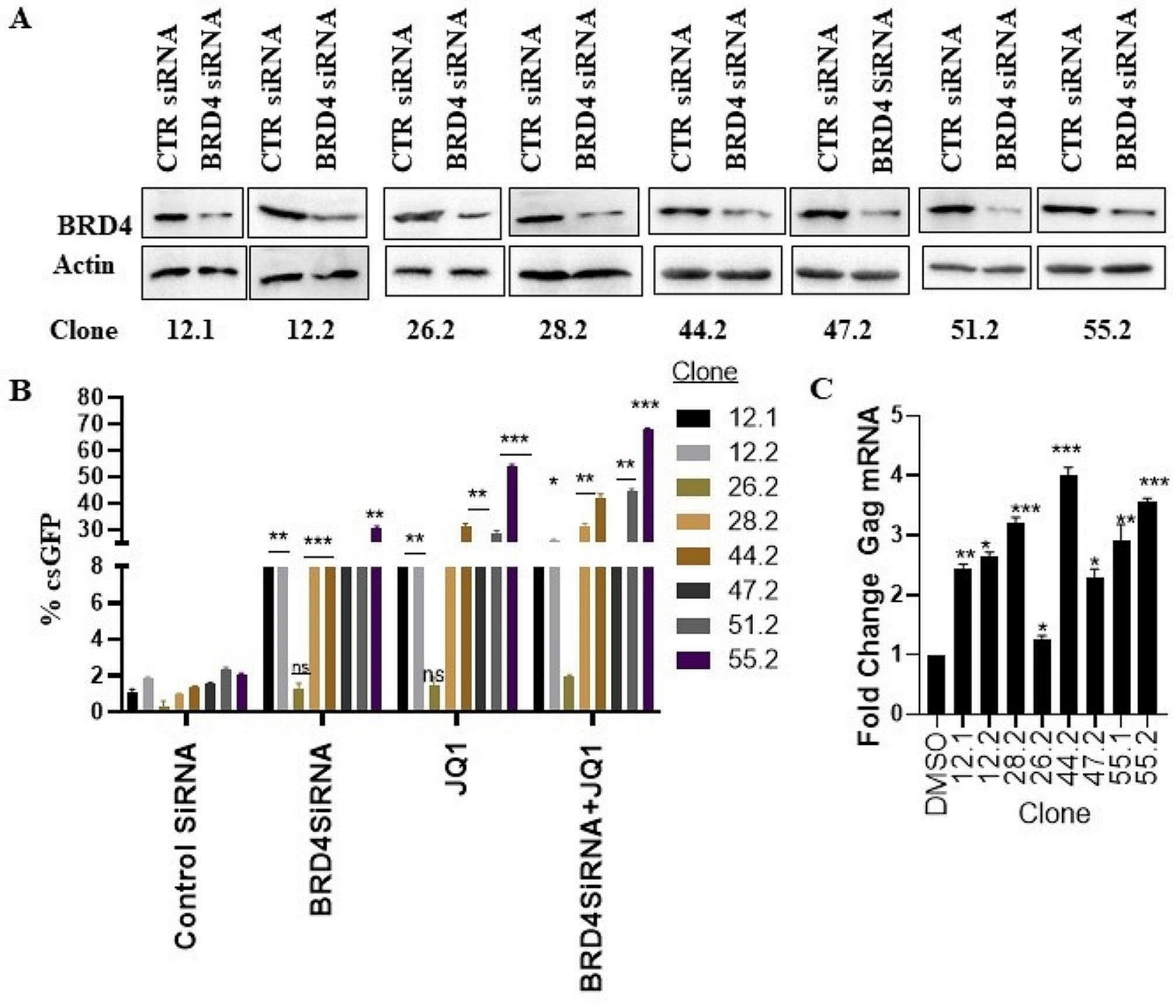
Knockdown of BRD4 reactivates latent HIV. (**A**) Western blot showing the knockdown of BRD4 in dTHP-1 latently infected clones with HIV. (**B, C**) HIV reactivation in cells from **A** after treatment with JQ1 was measured with FACS (**B**) or quantification of gag mRNA (**C**). Data are means, and error bars indicate SEM (*n = 3*). *, *p* < 0.05, **, *p* < 0.01; ***, *p* < 0.001, Student’s t test

Since JQ1 reversed HIV latency in differentiated THP-1 cells, we determined whether the knockdown of BRD4 had a similar effect. JQ1 is a BET family of proteins inhibitor which prevents BRD4-mediated transcriptional enhancement by interfering with the binding of BRD4 to acetylate histones [54–56]. BRD4 is both a positive and negative regulator of HIV-1 gene expression. Some studies suggest that BRD4 competes with Tat for binding PTEF-b during active transcription and that its over-expression may inhibit HIV-1 infection [57, 58]. Zhu et al. showed that the BRD4 inhibitor JQ1 increased HIV replication in both Hela cells and in a T cell line and that these effects were dependent on BRD4 [56]. We used siRNA to knock down BRD4 in several clones of dTHP-1 latently infected cell lines and achieved partial knockdown in all the eight clones tested (Fig. 4A). Cells were then reactivated with JQ1, and the extent of reactivation was measured with flow cytometry as a percentage of csGFP-positive cells as before. Knockdown of BRD4 without further reactivation, resulted in significantly increased HIV reactivation from 1 to 2% baseline to 8–30% depending on the clone. The extent of reactivation was less than JQ1 alone probably due to the partial knockdown (Fig. 4B). Importantly the JQ1-unresponsive cell line (clone 26.2) did not show any reactivation while the most responsive cell line (clone 55.2) showed the greatest reactivation upon BRD4 knockdown indicating that the JQ1 effects were mediated through BRD4. When we combined BRD4 siRNA and JQ1 there were marginal increases in reactivation, again indicating a BRD4 effect (Fig. 4B). Consistent with Fig. 2D, the knockdown of BDR4 increased HIV gag mRNA between 2.5- to 4-fold in 7 of 8 clones tested (Fig. 4C).

### Inhibition of BCL-2 selectively kills macrophages with reactivated HIV

**Fig. 5:**
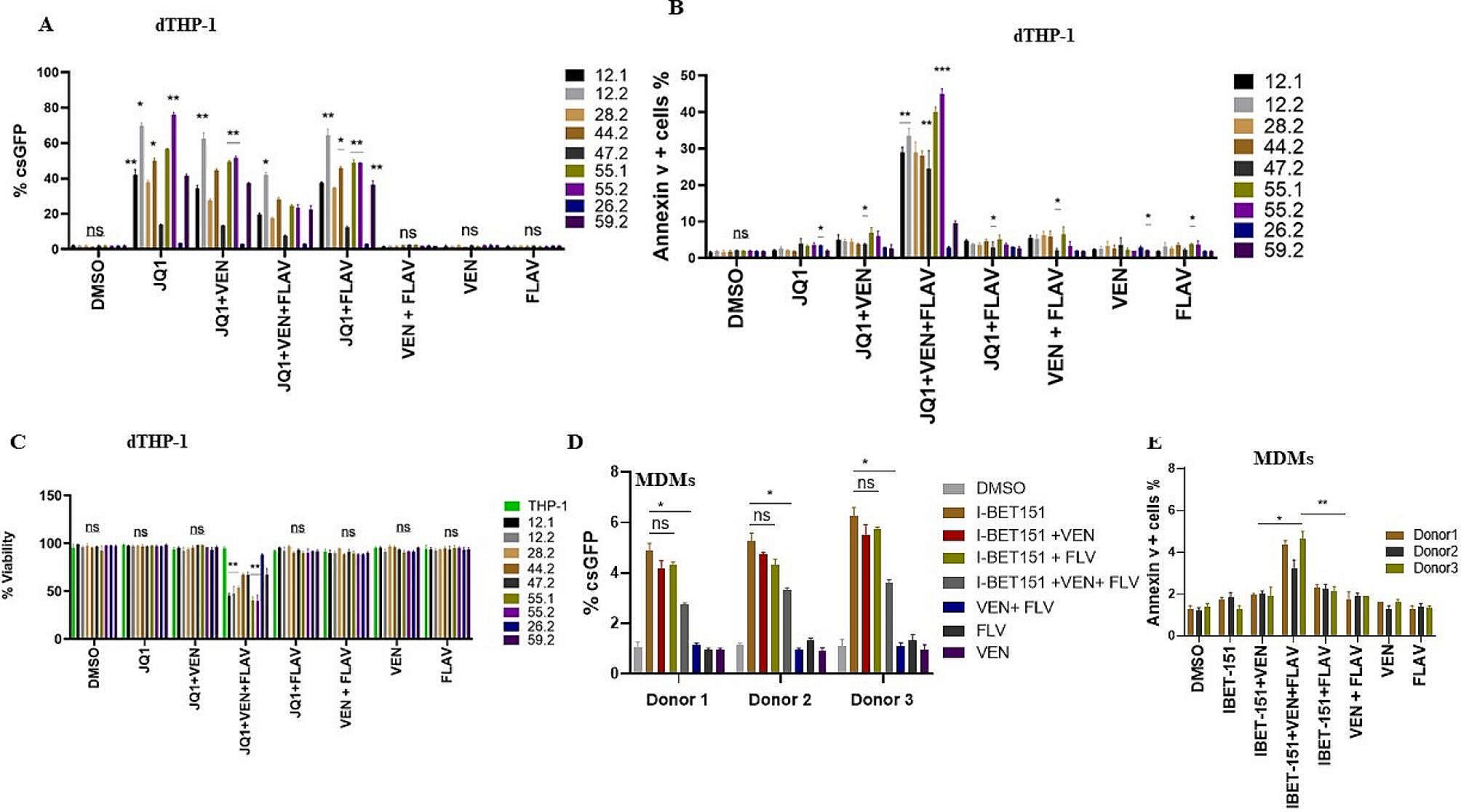
Inhibition of BCL-2 and CDK selectively kills macrophages with reactivated HIV. The combination of venotoclax and flavopiridol decreases the number of cells with reactivated HIV. (**A**) Latently infected dTHP-1 cells were treated with JQ1 for 48 h followed by treatment with or without BCL-2 antagonist venetoclax (50 nM) and CDK inhibitor flavopiridol (80 nM). (B) Differentiated THP-1 cell lines harboring reactivated HIV were treated with flavopiridol and venetoclax and then subjected to Annexin V-APC/PI staining and flow cytometry analyses. (**C**) Trypan blue exclusion cell viability assay. dTHP-1 cells were treated with Venetoclax and Flavopiridol after reactivation with JQ1. (**D**) Latently infected MDMs were treated with I-BET151 for 48 h followed by treatment with or without BCL-2 antagonist venetoclax (50 nM) and CDK inhibitor flavopiridol (80 nM), and csGFP + cells measure as before. (**E**) MDMs harboring reactivated HIV were treated with Flavopiridol and Venetoclax and then subjected to Annexin V-APC/PI staining and flow cytometry analyses. Data are means, and error bars indicate SEM (*n* = 3). *, *p* < 0.05, **, *p* < 0.01; ***, *p* < 0.001, Student’s t-test Data are means, and error bars indicate SEM (*n = 3*). ns, not significant, *, *p* < 0.01, **, *p* < 0.001; Student’s t test

The ‘kill’ part of the shock and kill approach has been a challenge because reactivated viruses often do not cause cell death. Likely, combination treatment with LRA and another agent that can kill cells with reactivated virus will be needed. This would be incredibly challenging in infected macrophages which are known to be resistant to apoptosis. Therefore, we determined whether dTHP-1 cells with the reactivated virus could undergo cell death when challenged with a BCL-2 antagonist venetoclax since it has been shown to selectively kill HIV-infected T cells [59]. THP-1 cells with latently integrated HIV were differentiated with PMA and then reactivated with JQ1 for 48 h followed by the treatment with venetoclax. The addition of venetoclax alone failed to reduce the proportion of JQ1-induced GFP + cells in dTHP-1 cells or I-BET151-induced reactivated cells in MDMs (Fig. 5A and D). Previous work has shown that the CDK inhibitor flavopiridol increases the pro-apoptotic ability of venetoclax [60], therefore we added flavopiridol together with venetoclax to determine the effect on csGFP-expressing cells in dTHP-1 cells and MDMs. A combination of venetoclax and flavopiridol resulted in a reduction of JQ1-induced csGFP expressing cells from 60 to 30% in dTHP-1 cells, and a 1.5 to 2-fold reduction in I-BET151-induced GFP + cells in MDMs (Fig. 5A and D). In trypan blue exclusion assays, a combination of JQ1, venetoclax, and flavopiridol did not affect uninfected dTHP-1 cells or infected cells with no reactivation (example clone 26.2) but led to 50% killing in cells with reactivated HIV (Fig. 5C). Using Annexin V flow cytometry, we confirmed that the reduction of csGFP cells was due to apoptosis in both dTHP-1 cells and MDMs (Fig. 5B and E,). Analysis of the ratio of csGFP + and Annexin V + cells showed that cells treated with the Venetoclax and Flavopiridol experienced the most cell death (Fig. S6). Together, the data show that the addition of venetoclax and flavopiridol results in the selective killing of macrophages harboring reactivated HIV.

## Discussion

In this study, we have established an HIV latency model in THP-1 cells that can be used to screen for latency-reversing agents in macrophages. Each stable cell line has one copy of HIV inserted at a different position in the genome, with differential responses to a variety of latency-reversing agents, most prominently, bromodomain inhibitors. While studies of HIV latency have focused mainly on CD4^+^ T cells, it is now clear that macrophages form an important part of the reservoir and may contribute to long-term latency and early viral rebound when ART is interrupted [61, 62]. However, compared to T cells, the cellular factors and mechanisms that control HIV replication, latency, and reactivation in macrophages are much less understood. Since primary macrophages are difficult to manipulate genetically and maintain in culture for prolonged periods, it is crucial to develop appropriate tools that can help uncover the mechanisms that control viral latency in macrophages. The interactions of HIV with replication enablers, restriction factors, and other cellular factors in differentiated THP-1 cells have proven similar in primary macrophages. Therefore, THP-1 cells provide a good model to study viral latency and reactivation in macrophages [63, 64]. We have also shown here each cell line has a different integration site. HIV latency occurs in extremely low frequency, and in patients who are virologically suppressed, the number of integration sites per cell could be one or less [65]. To mimic the heterogeneity of integration sites in vivo, we have used different cell lines that have different integration sites. The cell lines described in this model present a valuable tool to study the molecular mechanisms of HIV latency and identification of potential LRAs for macrophages.

A key finding of this study is that compared to resting CD4^+^ T cells well-known LRAs like PMA/ionomycin, bryostatin-1 and HDAC inhibitor Vorinostat had little reactivation potential in differentiated THP-1 cells or primary macrophages [66]. Interferon-gamma and TNF-α were mildly effective in these cells. This indicates that the LRAs that may be effective for CD4^+^ T cells are unlikely to have similar efficacy for cells of the monocyte lineage, making it imperative to discover new agents that can reactivate HIV from latency in macrophages. This is important because several of the HIV cure approaches such as shock and kill, immunotherapy, and chimeric antigen receptor T cells (CAR T cells), will all require some form of reactivation of the latent provirus. Of the many LRAs with different mechanisms of action that were tested, the bromodomain inhibitors JQ1 and I-BET151 showed the most significant reactivation in differentiated THP-1 and primary macrophages. Knockdown of BRD4, the target of JQ1 and I-BET151, resulted in reactivation indicating that the mechanism of action of these compounds worked through BRD4. More importantly, the clones that did not show reactivation upon knockdown also did not respond to the bromodomain inhibitors. The mechanisms determining why the differentiated THP-1 cells and primary macrophages were more sensitive to bromodomain inhibitors remain unclear and require further investigation.

It is also significant that different clones of dTHP-1 cells responded differently to the bromodomain inhibitors and other LRAs. For instance, whereas clone 26.2 with HIV integrated into the RERE gene showed virtually no reactivation irrespective of the LRA, clone 55.1 integrated into CCNL2 showed robust reactivation. In general, reactivatable clones (example 55.2, 12.1) were more likely to respond to pharmacological reactivation with LRAs and genetic manipulations such as the knockdown of BRD4. This observation is similar to what has been described in the Jurkat HIV cell line model (J-Lat) whereby a clone like J-Lat10.6 is sensitive to reactivation while J-Lat9.2 has limited reactivation regardless of the LRA employed. In the case of the J-Lat cell line, the difference in reactivation has been linked to different proviral integration sites [67]. In this study, although we suspect the integration site has a role in the response to LRAs, this has not been proven. Although HIV integrates in a non-random fashion mainly in intronic regions of actively transcribed genes [68], epigenetic silencing of provirus leads to the establishment and maintenance of HIV latency. This may occur through several factors such as availability of cellular or host transcription factors, chromatin organization of promoter, site, and orientation of integration [69].

In general, reactivation in the primary macrophages followed the same trend as in THP-1 cells in terms of response to the different LRAs, however, the overall response was subdued compared to differentiated THP-1 cells. A few reasons could account for this difference. The primary macrophages with latent HIV may be more difficult to reactivate with the compounds tested compared to differentiated THP-1 cells. The more likely reason, however, is that the infected macrophages evaluated in these experiments represented an oligoclonal mixture of proviral integration sites. Though digital droplet PCR showed that each MDMs were latently infected with one copy of HIV we could not isolate MDMs into single clones because they are terminally differentiated and could not be expanded in culture like the THP-1 cells. Future studies can examine this hypothesis using single-cell sequencing of latently infected macrophages. For MDMs, the consistent reactivation agents were I-BET151, LPS/interferon-gamma and Harmine, the DYRK1A inhibitor. We and others have shown that DYRK1A serves like an HIV restriction factor in macrophages [70], plays a role in T cell differentiation [71], and may help enforce latency in resting CD4 + T cells. Therefore, the role of DYRK1A in resting T cells and macrophages, in terms of latency and reactivation deserves further investigation. Taken together, the finding that most well-known LRAs had limited reactivation potential in dTHP-1 and MDM, shows that more work is needed to identify reactivation agents for this cell type.

Unlike T cells, HIV-infected macrophages are known to be resistant to cell death mechanisms such as apoptosis. Several mechanisms have been implicated in resistance to cell death including viral proteins such as gp120 and Nef as well as cellular factors like miRNA [72]. Recently, Wang et al. showed that reactivated cells, including macrophages, could undergo cell death through the process of pyroptosis under specific conditions [73]. 

Thus, we examined whether reactivated dTHP-1 cells with reactivated viruses could undergo cell death. We found that up to 50% of cells reactivated with any of the LRAs could die when cells were treated with flavopiridol, affirming the principle that macrophages with a newly reactivated virus (shock) could undergo cell death, not by viral cytopathic effect but by the addition of a ‘kill’ agent that can trigger cell death through additional mechanisms. Increasingly, more mechanisms for killing cells with reactivated viruses are being discovered for T cells, but these studies need to be performed in macrophages as well.

## Conclusion

The model system described here provides a new platform for screening for novel compounds that could reactivate latent HIV in macrophages, made more critical by the fact that most of the known LRAs are ineffective in macrophages. These cells could also be adapted to screen for compounds for the block and lock approach once more effective reactivation agents are found for macrophages. Our findings show that it is possible to develop a system of viral latency in macrophages that is amenable to genetic manipulation that can help uncover the mechanisms of latency in macrophages and provides a critical reagent for scientists to discover more latency-reversing agents for macrophages.

### Electronic supplementary material

Below is the link to the electronic supplementary material.

Supplementary Material 1

## Data Availability

No datasets were generated or analysed during the current study.
