# Supplementary material for: A macrophage-cell model of HIV latency reveals the unusual importance of the bromodomain axis
Source: Virol J. 2024 Apr 5;21:80. doi: 10.1186/s12985-024-02343-9 (PMC10996205; doi:10.1186/s12985-024-02343-9)
Supplement: Supplementary file 1 — Supplementary Material 1 [file 12985_2024_2343_MOESM1_ESM.docx]

**Supplementary Materials**

**A macrophage-cell model of HIV latency reveals the unusual importance of the bromodomain axis**

Javan Kisaka ^1^, Daniel Rauch^1^, Malachi Griffith ^1,2^ and George Boateng Kyei ^1,3,4,5,^ *

^1^Department of Medicine, Washington University School of Medicine in St. Louis, Missouri 63110, USA; javan@wustl.edu

^2^McDonnell Genome Institute, Washington University School of Medicine in St. Louis, Missouri 63108, USA; [mgriffir@wustl.edu](mailto:mgriffir@wustl.edu)

^3^Department of Molecular Microbiology, Washington University School of Medicine in St. Louis, Missouri 63110, USA; g.kyei@wustl.edu

^4^Department of Virology, Noguchi Memorial Institute for Medical Research, College of Health Sciences, University of Ghana, Accra, Ghana; g.kyei@wustl.edu

^5^Medical and Scientific Research Center, University of Ghana Medical Center, Accra, Ghana; g.kyei@wustl.edu

***** Correspondence: g.kyei@wustl.edu; Tel. (314) 454-8293; Fax. 314 454-5392

**Supplementary Materials:**

**Figure S1**


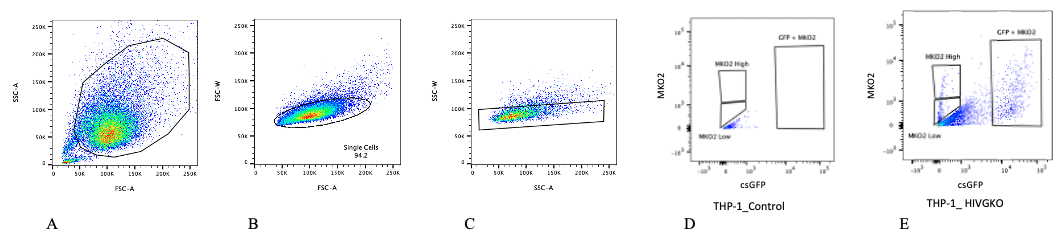


**Gating strategy to sort out latently infected, productive and uninfected cells.** (A) The gate setting for live cells using side (SSC-Area) and forward scatter (FSC-Area) analysis. (B, C) Successive gating on the singlets FSC-area vs. FSC-width and SSC-width vs. SSC-area. (D, E) Gate on GFP/FITC-area positive to sort productively infected cells, or on GFP/FITC-are negative vs. MKO2/PE-Area^positive^ to sort latently infected cells (MKO2^+^/GFP^-^).

**Figure S2**

**
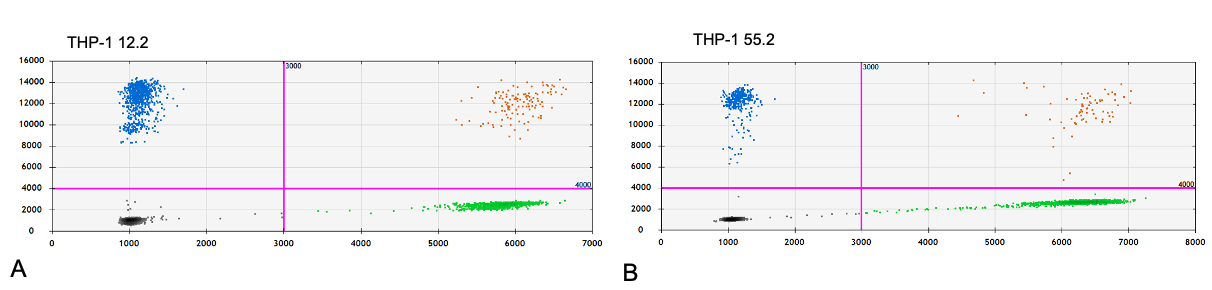
**

**Viral copy number determination using digital droplet PCR. A-B.** Sample clones represent a 2-D fluorescence amplitude plot of replicate wells of a copy number sample duplexed with HIV-1 *5-LTR* and *RPP30* assays. The blue cluster represents the droplets that are positive for *HIV 5’ LTR* only, the black cluster on the plot represents the negative droplets, the orange cluster represents the droplets that are positive for both *5’ LTR* and *RPP30* and the green cluster represents the droplets that are positive for *RPP30* reference only.

**Figure S3**

**A schematic overview of the adaptor/inker-mediated amplification of HIV provirus integration sites.**

**Figure S4**

**Intergration site analysis.** (A) HIV-1 integration sites for the latently infected THP-1 cells. (B) DNA agarose gel of the PCR product of 150 bp to confirm the integration sites. (C) mRNA expression levels of 4 of the integration site genes in dTHP-1 cells. (D) Western blot showing expression of 3 of the genes in dTHP-1 cells and MDMs.

**Table S1:** Percent of mKO2+ and csGFP+ after reactivation with JQ1 in Figure 2A

| **Clone #** | **%mKO2+ after reactivation** | **%GFP+, mKO2+** |
| --- | --- | --- |
| **12.1** | 76.3 | 22.64 |
| **12.2** | 34.1 | 65 |
| **26.2** | 97.6 | 1.4 |
| **28.2** | 67.5 | 30.75 |
| **44.2** | 67.45 | 31.65 |
| **55.1** | 53.3 | 45.4 |
| **55.2** | 23.8 | 74.1 |
| **69.2** | 94.4 | 3.5 |
| **31.1** | 93.1 | 6.3 |

**Figure S5**

Gating strategy to sort out latently infected, productively, and uninfected MDMs. A similar process as in Fig S1 was used.


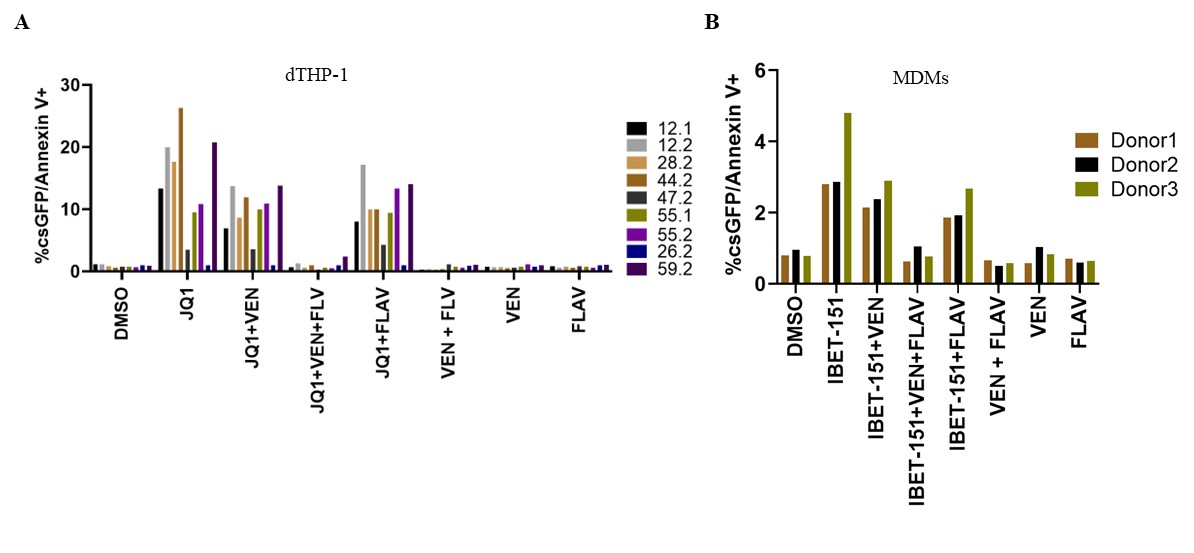
**Figure S6**

The ratio of percent csGFP to Annexin V+ cells from dTHP-1 cells (derived from Figures 5A and 5B) and MDMs (derived from Figures 5D and 5E).

**Table S2: List of primers used**

| RPP30 forward | GAT TTG GAC CTG CGA GCG |
| --- | --- |
| RPP30 reverse | GCG GCT GTC TCC ACA AGT |
| RPP30 probe | VIC-CTG ACC TGA AGG CTC T-MGB |
| Ψ Forward (692-711) | CAGGACTCGGCTTGCTGAAG |
| Ψ Reverse (797-775) | GCACCCATCTCTCTCCTTCTAGC |
| Ψ Probe | FAM-TTTTG GCGTACTCACCAGT-MGB |
| MH535 Forward | AAC TAG GGA ACC CAC TGC TTA AG |
| ENV Reverse | GTCTGGCCTGTACCGTCAGC |
| Gag Forward | ACATCAAGCAGCCATGCAAAT |
